# Prevalence of *pks* + bacteria and enterotoxigenic *Bacteroides fragilis* in patients with colorectal cancer

**DOI:** 10.1186/s13099-022-00523-y

**Published:** 2022-12-28

**Authors:** Manon Oliero, Roy Hajjar, Thibault Cuisiniere, Gabriela Fragoso, Annie Calvé, François Dagbert, Rasmy Loungnarath, Herawaty Sebajang, Frank Schwenter, Ramses Wassef, Richard Ratelle, Éric De Broux, Carole S. Richard, Manuela M. Santos

**Affiliations:** 1 Nutrition and Microbiome Laboratory, Institut du cancer de Montréal, Centre de recherche du Centre Hospitalier de l’Université de Montréal (CRCHUM), 900 Rue Saint Denis, Montréal, QC H2X 0A9 Canada; 2 Department of Surgery, Faculty of Medicine, Université de Montréal, 2900 Boulevard Édouard-Montpetit, Montréal, QC H3T 1J4 Canada; 3 Digestive Surgery Service, Department of Surgery, Centre Hospitalier de l’Université de Montréal (CHUM), 1000 Rue Saint-Denis, Montréal, Québec H2X 0C1 Canada; 4 Department of Medicine, Faculty of Medicine, Université de Montréal, 2900 Boulevard Édouard-Montpetit, Montréal, QC H3T 1J4 Canada

**Keywords:** Colibactin, Enterotoxigenic *Bacteroides fragilis*, Tumorigenesis, Colorectal cancer

## Abstract

**Background:**

Colorectal cancer (CRC) is the third most diagnosed cancer and the second most common cause of cancer deaths worldwide. CRC patients present with an increase in pathogens in their gut microbiota, such as polyketide synthase-positive bacteria (*pks* +) and enterotoxigenic *Bacteroides fragilis* (ETBF). The *pks* + *Escherichia coli* promotes carcinogenesis and facilitates CRC progression through the production of colibactin, a genotoxin that induces double-strand DNA breaks (DSBs). ETBF is a procarcinogenic bacterium producing the *B. fragilis toxin* (*bft*) that promotes colorectal carcinogenesis by modulating the mucosal immune response and inducing epithelial cell changes.

**Methods:**

Fecal samples were collected from healthy controls (N = 62) and CRC patients (N = 94) from the province of Québec (Canada), and a bacterial DNA extraction was performed. Fecal DNA samples were then examined for the presence of the *pks* island gene and *bft* using conventional qualitative PCR.

**Results:**

We found that a high proportion of healthy controls are colonized by *pks* + bacteria (42%) and that these levels were similar in CRC patients (46%). *bft* was detected in 21% of healthy controls and 32% of CRC patients, while double colonization by both *pks* + bacteria and ETBF occurred in 8% of the healthy controls and 13% of the CRC patients. Most importantly, we found that early-onset CRC (< 50 years) patients were significantly less colonized with *pks* + bacteria (20%) compared to late-onset CRC patients (52%).

**Conclusions:**

Healthy controls had similar levels of *pks* + bacteria and ETBF colonization as CRC patients, and their elevated levels may place both groups at greater risk of developing CRC. Colonization with *pks* + bacteria was less prevalent in early-compared to late-onset CRC.

**Supplementary Information:**

The online version contains supplementary material available at 10.1186/s13099-022-00523-y.

## Background

The composition and function of the gut microbiome have been shown to potentially influence the initiation and progression of colorectal cancer (CRC) [1]. Patients with CRC have an unbalanced gut microbiome, or dysbiosis, which is characterized by a decrease in beneficial bacteria and an increase in pathobionts, such as colibactin-producing *Escherichia coli* and enterotoxigenic *Bacteroides fragilis* (ETBF) [2].

While gut microbiota contains commensal *E. coli* strains, some strains may carry a pathogenic potential [3]. The *pks* genomic island contains the colibactin (*clb*) gene cluster, which encodes the genes required for colibactin synthesis [4]. Colibactin is a genotoxin that causes inter-strand cross-links (ICLs) [5] and double-strand DNA breaks (DSBs), cell cycle arrest, senescence, and chromosomal abnormalities in mammalian cells [6]. Murine models of *pks* + *E. coli* mono-colonization [7, 8] and colonization of adenomatous polyposis coli multiple intestinal neoplasia (*Apc*^*Min/*+^*)* mice with colibactin producing *E. coli* [9] revealed a causal link between the presence of colibactin and intestinal tumorigenicity. Other *Enterobacteriaceae* species, such as *Klebsiella*, inherited the *pks* island and some genes of the cluster from horizontal transfer and can also produce colibactin [10, 11]. Colonization with colibactin-producing bacteria in humans occurs mainly during early life [12], and the presence of the phylogroup of *pks* + *E. coli* is steadily increasing worldwide [13, 14].

*Bacteroides* strains such as EBTF have also been associated with CRC. ETBF which produces *Bacteroides fragilis* toxin (*bft*), has been shown to contribute to colon carcinogenesis [15] through induction of colonocyte proliferation [16], inhibition of apoptosis and promotion of proinflammatory signaling [17, 18]. Accordingly, ETBF colonization in a murine model of colitis-induced CRC increased the number of tumours [19], while in the *Apc*^*Min/*+^ CRC mouse model, it promoted the development of colon adenomas [20], further confirming its carcinogenic potential.

In this study we assessed the prevalence of *pks* + bacteria and ETBF in a cohort of 94 CRC patients and 62 healthy individuals from the province of Québec, Canada.

## Methods

### Patient recruitment and sample collection

Patients with CRC and healthy individuals were recruited at the Centre hospitalier de l’Université de Montréal (CHUM) (Additional file 1: Table S1). Individuals with inflammatory bowel disease (IBD), polyps or antibiotic treatment 6 months prior to sampling were excluded from the control group. Participants were requested to provide a fresh fecal sample collected at home following the International Human Microbiome Standards procedure [21]. Samples were collected in hermetic containers with an anaerobic sachet (BD BBL^™^ GasPak^™^ anaerobic indicator, BD, ON, Canada) and stored at −80 °C upon arrival at the laboratory within 24 h of sampling.

### DNA extraction and polymerase chain reaction

Total DNA was extracted from human fecal samples with the PowerSoil^®^ DNA Extraction Kit (Qiagen Inc., Toronto, ON, Canada) and polymerase chain reaction (PCR) was performed using PowerUp^™^ SYBR^™^ Green Master Mix (Thermo Fisher Scientific, Waltham, MA, USA) in the RG 3000A R PCR machine (Qiagen Inc.) using the following cycling conditions; 50 °C for 2 min, 95 °C for 2 min, 38 cycles of 15 s at 95 °C, followed by 1 min at 60 °C. Simultaneous amplification of *colibactin A* gene (*clbA*) and *E. coli* 16S rRNA were done with the following primers for *clbA*: Fw 5ʹ-CTCCACAGGAAGCTACTAAC-3ʹ, Rv 5ʹ-CGTGGTGATAAAGTTGGGAC-3ʹ [4] and *Ecoli 16S*: Fw 5ʹ-GTTAATTTTGCTCATTGA-3ʹ, Rv 5ʹ-ACCAGGGTATCTAATCCTGTT-3ʹ[22], with a 1:1:1:1 ratio. For the detection of ETBF, we performed a simultaneous PCR of the *bft* gene and *B. fragilis* 16S rRNA with the following primers for *bft*: Fw 5ʹ*-GAACCTAAAACGGTATATGT-3’, Rv 5*ʹ*-GTTGTAGACATCCCACTGGC-3*ʹ [8] and *Bfr:* Fw 5ʹ*-*CTGAACCAGCCAAGTAGCG-3ʹ, Rv 5ʹ-CCGCAAACTTTCACAACTGACTTA-3ʹ [23], with a 5:5:1:1 ratio. We used the *E. coli* NC101 strain (EcNC101 (a gift from Dr. Christian Jobin, Cancer Microbiota & Host Response, UF Health Cancer Center, University of Florida)) as a positive control for the presence of the *pks* island, and the ETBF strain (a gift from Dr. Cindy Sears, Johns Hopkins University School of Medicine [8]) as the positive control for the *bft* gene. The PCR products were then visualized on a 1.8% agarose gel containing Eco-stain plus (Bio Basic Inc., Markham, ON, Canada). The expected product sizes were: 330 bp for *E. coli* 16S rRNA; 300 bp for *clbA*; 230 bp for *B. fragilis* 16S rRNA; and 370 bp for *bft*.

### Statistics

All data were analyzed using GraphPad Prism (Version 5.0, GraphPad Software, San Diego, CA, USA). χ2 tests were used to compare categorical variables, unless expected frequencies were  < 5, in which case Fisher’s exact test was used. *P* < 0.05 were considered statistically significant.

## Results and discussion

**Fig. 1:**
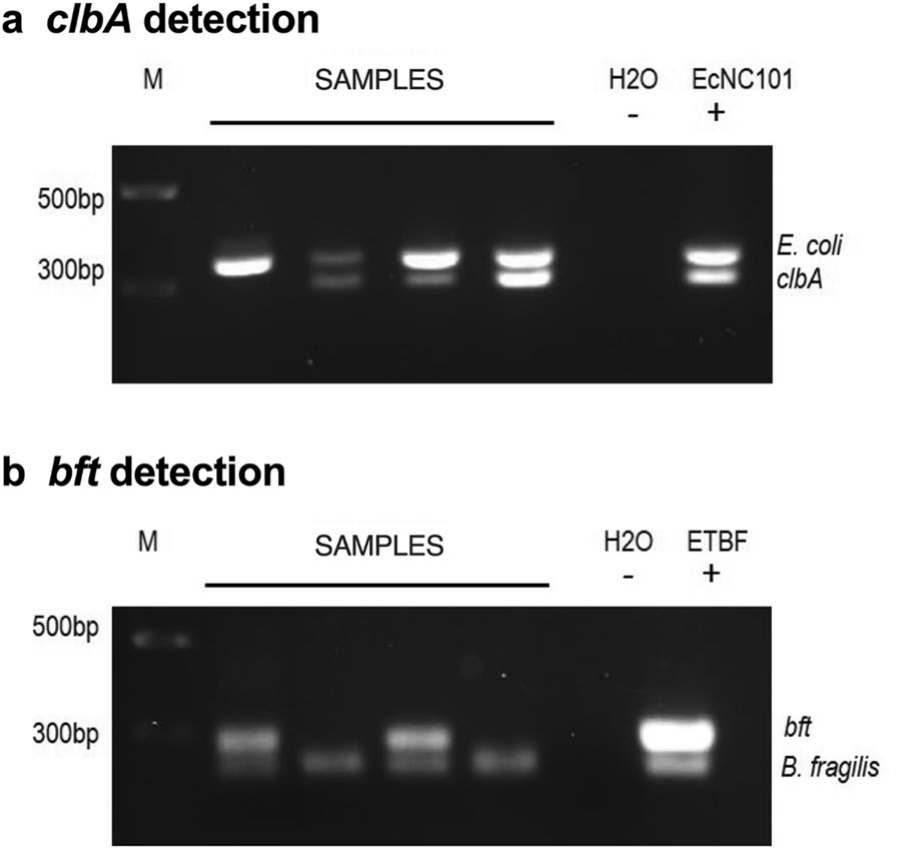
*clbA* and *bft* detection.** a** *clbA* and **b** *bft* presence in DNA extracted from fecal samples were detected using conventional qualitative PCR. M: marker; negative (−) control: water (H_2_O); positive control ( +): DNA extracted from *pks* + EcNC101 or ETBF bacteria

**Fig. 2:**
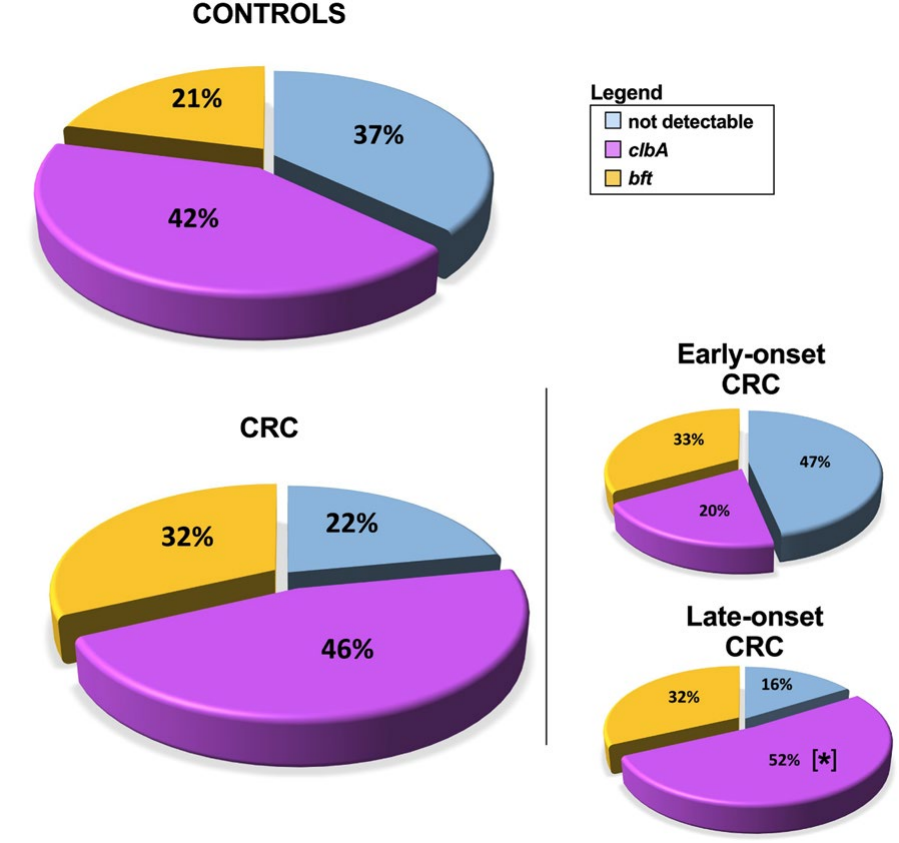
Prevalence of *pks* + and ETBF in the cohort. Controls – healthy participants; CRC – colorectal cancer patients. * *P* < 0.05, Fisher’s exact test

The presence of colibactin-producing bacteria in stool samples collected from participants (Additional file 1: Table S1) was detected by PCR using specific primers targeting the *clbA* gene encoded in the *pks* island, required for colibactin production [4]. In addition, as a positive control for the PCR reaction, primers universal for all strains of *E. coli* were used [22] (Fig. 1a). We found that 42% of healthy donors and 46% of CRC patients were colonized by a *pks* + bacteria (Fig. 2, Table 1). Interestingly, *pks* + bacteria were more prevalent in late-onset (40 out of 79; 52%) compared to early-onset CRC (3 out of 15; 20%; *P* < *0.05*) (Fig. 2, Table 1). Overall, the levels of *pks* + bacteria colonization in our CRC patients were within the range previously reported in literature with 68% [8] and 66.7% [7] in two cohorts from the USA, 56.4% in Sweden [24], 43% in Japan [25], 23% in Iran [26], and 16.7% in Malaysia [27]. As for the healthy population, they approached levels reported in a Japanese cohort (46%) [25], whereas lower levels were found in other healthy cohorts: 22% [8] and 20.8% [7] in the USA; 18.5% in Sweden [24]; 7.1% in Iran [26] and 4.35% in Malaysia [27]. These disparities in prevalence around the world could be attributed to dietary differences. For example, the so-called Western diet [28] has been linked to a higher incidence of colorectal cancer containing *pks* + *E. coli* [29]. Our study indicates that colibactin-producing bacteria are less prevalent in early-onset compared to late-onset CRC, although this finding should be confirmed in larger cohorts. While this could indicate that colibactin-producing bacteria may not be involved in the etiology of early-onset CRC, we cannot rule out that *pks* + *E. coli* and other colibactin-producing bacteria may have been present during childhood and subsequently eliminated, with the effects of early mutagenic exposure manifesting later in life [30].

**Table 1:** Prevalence of *clbA* and *bft* in controls and CRC patients

	Total (156)	*clbA* +	*p*-value	*bft* +	*p*-value
Cohort (%)					
Controls	62 (40)	26 (42)	N.s	13 (21)	N.s
CRC	94 (60)	43 (46)		30 (32)	
Controls					
Sex (%)					
Female	33 (53)	11 (33)	N.s	6 (18)	N.s
Male	29 (47)	15 (52)		7 (24)	
CRC					
Onset (%)					
Early	15 (16)	3 (20)	0.046	5 (33)	N.s
Late	79 (84)	40 (51)		25 (32)	
Sex (%)					
Female	38 (40)	14 (37)	N.s	12 (32)	N.s
Male	56 (60)	29 (52)		18 (32)	
Location (%)					
Proximal	26 (28)	14 (54)	N.s	7 (27)	N.s
Distal	68 (72)	29 (43)		23 (34)	
Stage (%)					
I	13 (14)	5 (38)	N.s	1 (8)	N.s
II	22 (23)	13 (59)		7 (32)	
III	48 (51)	21 (44)		16 (33)	
IV	10 (11)	4 (40)		4 (40)	

Other possible explanations for the increasing incidence rate of CRC in the younger population [31] could be related to early exposures to a deleterious lifestyle, environmental pollutants, a western diet [32], diets high in sugar [33], metabolic diseases during adolescence [34] or other components of the gut microbiota, such as the genera *Fusobacterium* and *Flavonifractor* [35].

To detect the presence of ETBF among the cohort, PCR using specific primers targeting the *bft* gene [8] was performed. Additionally, as a positive control for the PCR reaction, primers universal for *B. fragilis* strains [23] were used (Fig. 1b). *Bft* was detected in 21% of healthy donors and 32% of CRC patients (Fig. 2, Table 1). Overall, the levels of ETBF colonization in our CRC patients were within the range previously reported from other cohorts with 6.1% in Japan [36], 31.6% [37] and 47% [38] in two Iranian cohorts, 38% in Turkey [16], 49.3% in New Zealand [39], and 60% in the USA [8]. Regarding healthy controls, prevalence in our cohort was higher than those reported in the Turkish cohort (12%) [16], and in two Iranian cohorts (3.8% and 8.3%) [37, 38], whereas higher levels were reported in a cohort from the USA (30%) [8].

Finally, double colonization with *pks* + bacteria and ETBF was detected in 8% of healthy individuals and 13% of CRC patients (Table 2). In a US cohort, higher levels of double colonization with *pks* + bacteria and ETBF were detected in the healthy population (22%), with even higher levels reported in patients with familial adenomatous polyposis (FAP) (52%) [8]. Of note, the presence of both *pks* + bacteria and ETBF may lead to higher colonic inflammation and tumorigenesis [8].

**Table 2:** Prevalence of double colonization in controls and CRC patients

	Total (156)	*clbA* + */bft* +	*p*-value
Cohort (%)			
Controls	62 (40)	5 (8)	N.s
CRC	94 (60)	12 (13)	
Controls			
Sex (%)			
Female	33 (53)	1 (3)	N.s
Male	29 (47)	4 (14)	
CRC			
Onset (%)			
Early	15 (16)	0 (0)	N.s
Late	79 (84)	12 (15)	
Sex (%)			
Female	38 (40)	2 (5)	N.s
Male	56 (60)	10 (18)	
Location (%)			
Proximal	26 (28)	3 (12)	N.s
Distal	68 (72)	9 (13)	
Stage (%)			
I	13 (14)	0 (0)	N.s
II	22 (23)	6 (27)	
III	48 (51)	4 (8)	
IV	10 (11)	2 (20)	

## Conclusion

The prevalence of colibactin-producing bacteria and ETBF in CRC patients from our cohort was within the range reported in other studies. Nevertheless, we found that healthy controls had higher prevalence of *pks* + bacteria and ETBF when compared to most of the other cohorts. However, when comparing different reports, it should be taken into account that the type of tissue (mucosal *vs.* fecal samples) and measurement techniques (cultured *vs.* direct PCR) used to determine the prevalence of pro-carcinogenic bacteria may account for some of the variations between reported results. In any case, as these healthy individuals may be at a higher risk of developing CRC due to the potentially elevated levels of *pks* + bacteria and ETBF, it is critical to propose adapted dietary and medical interventions to regulate the abundance of these bacteria**.** A novel result of our study is the finding of a low prevalence of *pks* + bacteria in early-onset compared to late-onset CRC. Further studies are needed to understand the role of colibactin-bacteria in early-onset CRC.

## Supplementary Information

**Additional file 1: Table S1**. Demographic and clinical characteristics of the cohort.

## Data Availability

The authors declare the data used to support the findings of this study are available from the corresponding author upon request.

## Abbreviations

*Apc*^*Min/*^^+^: Adenomatous polyposis coli multiple intestinal neoplasia
*Bft*: *Bacteroides fragilis* toxin
*Clb*: Colibactin
clbA: *colibactin* *A* *gene*
CRC: Colorectal cancer
DSBs: Double-strand DNA breaks
ETBF: Enterotoxigenic *Bacteroides fragilis*
FAP: Familial adenomatous polyposis
IBD: Inflammatory bowel disease
ICLs: Inter-strand cross links
PCR: Polymerase chain reaction
*Pks*: Polyketide synthase
