# Supplementary material for: Prevalence of pks + bacteria and enterotoxigenic Bacteroides fragilis in patients with colorectal cancer
Source: Gut Pathog. 2022 Dec 28;14:51. doi: 10.1186/s13099-022-00523-y (PMC9798702; doi:10.1186/s13099-022-00523-y)
Supplement: Supplementary file 1 — Additional file 1: Table S1. Demographic and clinical characteristics of the cohort. [file 13099_2022_523_MOESM1_ESM.docx]

**Additional Table 1**. Demographic and clinical characteristics of the cohort

|  | Heathly controls (N=62) | CRC patients (N=94) | p-value |
| --- | --- | --- | --- |
| Age (median, [range]) | 58, [24-78] | 67, [22-91] | *0.0002* |
| Sex, n (%)  Female  Male | 33 (53)  29 (47) | 38 (40)  56 (60) | *0.116* |
| BMI, kg/m^2^(mean ± SD) | 27.5 ± 4.9 | 28.3 ± 6.1 | *0.390* |
| Location, n (%)  proximal  distal | n.a.  n.a. | 26 (28)  68 (72) |  |
| Stage, n (%)  I  II  III  IV | n.a.  n.a.  n.a.  n.a. | 13 (14)  22 (23)  48 (51)  10 (11) |  |

Age, BMI: Student’s t-test

Sex*:*  Chi-squared test

CRC, colorectal cancer; BMI, body mass index; n.a., not applicable; SD, standard deviation.
